# Pharmacist Services in the Opioid Crisis: Current Practices and Scope in the United States

**DOI:** 10.3390/pharmacy7020060

**Published:** 2019-06-13

**Authors:** Tanvee Thakur, Meredith Frey, Betty Chewning

**Affiliations:** 1 Social and Administrative Sciences Division, School of Pharmacy, University of Wisconsin-Madison, Madison, WI 53705, USA; betty.chewning@wisc.edu; 2 School of Pharmacy, University of Wisconsin-Madison, Madison, WI 53705, USA; mlfrey@wisc.edu

**Keywords:** pharmacist services, opioid, communication, naloxone, misuse, disposal, safety, counseling

## Abstract

**Introduction:** Pharmacist roles promoting safe opioid use are recognized in literature and practice. Pharmacists can offer services such as counseling on opioid risks, naloxone dispensing, education on opioid storage and disposal, prescription drug monitoring program (PDMP) utilization, opioid deprescribing, and providing resources for addiction treatment to help mitigate the opioid crisis. **Objective:** This commentary seeks to describe current and potential roles for pharmacists to combat the United States opioid crisis and identify key factors affecting service provision. **Methods:** The paper summarizes evidence-based studies describing current pharmacist roles and services, factors affecting service implementation, and strategies to further improve pharmacist roles and services related to promoting safe opioid use for patients. **Results:** Pharmacists recognize their roles and responsibilities to counsel patients on opioid risks, dispense naloxone, educate on opioid storage and disposal, utilize prescription drug monitoring programs (PDMPs), offer opioid deprescribing, and provide resources for addiction treatment. However, pharmacists express low confidence, time, and training as barriers to service provision. This suggests a need for structured training, resources, and organizational support for pharmacists to improve confidence and participation in such services. **Conclusions:** Although pharmacists are aware of roles and responsibilities to help reduce the opioid crisis, more training, education, organizational support and resources are needed to increase their ability to embody these roles.

## 1. Introduction

Opioid medications have the potential to cause dependence characterized by a strong desire to take opioids, persistent opioid use despite harmful consequences, increased tolerance, a physical withdrawal reaction when opioids are discontinued, and potential fatal overdose [1]. In 2016, 34 million people globally reported using opioids consistently and about 19 million people used opioids at least once [2]. Deaths attributed to opioid overdose contribute up to half of all drug-related deaths globally [2]. There are effective treatments for opioid use disorders, and yet less than 10% of people who would benefit from treatment are receiving it [2].

In the United States (U.S.), prescription opioids specifically contribute largely to the current opioid crisis, with more than 40% of all opioid overdose deaths in 2016 involving a prescription opioid, and more than 46 people dying every day from overdoses involving prescription opioids [3]. From 1999–2016, more than 200,000 people died from overdose related to prescription opioids alone [4]. Approximately 1000 individuals are treated in emergency departments (ED) each day for prescription opioid misuse [5]. Deaths related to opioid overdose have increased exponentially and will likely continue to grow, making this an important national issue in the U.S. and the focus of this commentary.

Although many individuals and organizations are involved in combating the opioid crisis nationwide, healthcare professionals are primarily responsible for appropriate opioid prescribing and promoting safe opioid use [6]. Pharmacists are especially well-positioned to contribute to safe opioid use due to their role dispensing opioid prescriptions and accessibility to patients [7,8]. Pharmacists utilize prescription drug monitoring programs (PDMP) to help prevent diversion of opioids and detect inappropriate prescribing, and can monitor and recognize signs of opioid misuse [8]. PDMP is a state-based electronic database accessed by prescribers and pharmacists to track controlled substance prescribing and dispensing [9]. Specific information collected from the pharmacy includes what controlled substances were dispensed, how much, to whom, and by whom [9]. Pharmacists can extend patient counseling responsibilities by educating patients on risks associated with opioid use, proper storage and disposal of medication, and the consequences of sharing medications with another person [10]. Additionally, many pharmacists can distribute naloxone, an opioid antagonist effective in reversing respiratory depression from opioid use, enabling a family member, friend, or potential bystander to prevent death associated with opioid overdose [11]. Pharmacists are knowledgeable about addiction treatment options and can connect patients to resources within the community [8]. Although pharmacists are equipped to provide these services to help mitigate the opioid crises, current literature documents that pharmacists’ breadth of opportunities for services is far from fully realized. Underlying factors need to be considered to help pharmacist meet their potential to provide services related to opioid use.

## 2. Objectives

This commentary seeks to describe current and potential roles for pharmacists to reduce the opioid crisis, identify key factors affecting service provision, and suggest strategies for improvement.

## 3. Methods

An exhaustive search of PubMed and Cinhal databases was conducted to identify evidence-based studies describing current pharmacist roles and services, factors affecting service implementation, and strategies to further pharmacist roles and services related to promoting safe opioid use for patients. The following search terms and combinations were used: opioids, pharmacy, pharmacist, safety, counseling, disposal, naloxone, PDMP, and deprescribing. Only studies conducted in the United States were included. The available evidence was summarized into six main categories based on pharmacist roles: counseling on opioid risks, naloxone dispensing, opioid storage and disposal, PDMP utilization, opioid deprescribing, and addiction treatment resources.

## 4. Results

### 4.1. Counseling on Opioid Risks and Safety

A nationally distributed case vignette survey of primary care physicians (PCPs), pain specialists, and pharmacists, along with nested chart reviews and surveys of patients with chronic pain revealed that prescribers and pharmacists often omit key messages during patient counseling regarding safe opioid use [12]. Among pharmacists, safety counseling is generally limited to informing patients of potential side effects [12]. The omission of information provided may be due to limited self-efficacy among pharmacists in communicating with patients regarding prescription drug abuse and misuse. Pharmacists cited common barriers to communication as lack of confidence, training, and time [13]. Barriers to discussing opioid therapy were similar to those cited for discussing drug abuse and pain with patients, including lack of confidence, training and inadequate access to health information [7]. These barriers are compounded by uncertainty among pharmacists and patients regarding pharmacists’ roles in opioid safety. Patients and pharmacists perceived pharmacists to be responsible for medication safety, yet the majority of pharmacists were uncomfortable dispensing opioids and felt they were “policing” opioid prescriptions [14]. Overall, there is a paucity of literature describing current counseling practices pharmacists use to educate patients about opioid risks, such as dependence and overdose, or mechanisms to promote safe opioid use.

### 4.2. Naloxone Dispensing

Increased state and national legislative and regulatory initiatives are partly due to greater recognition and acceptance of naloxone use by the general public, people who use drugs (PWUD), and healthcare professionals such as pharmacists. About half of states have increased funding to expand patient access to naloxone, pharmacologic treatment options for PWUD, and guidelines for safe opioid prescribing [15]. Standing orders have been implemented on a national level, and allow for naloxone dispensing by pharmacists or other healthcare professionals without a patient-specific prescription [16]. Pharmacists have successfully utilized standing orders to increase patient access to and distribution of naloxone in many pharmacy settings [17]. State and national policies have facilitated the expansion of pharmacist roles, enabling pharmacist to be a key resource in opioid overdose prevention [18]. Pharmacists utilize these policies and standing orders to identify patients who are at risk of overdose and would benefit from naloxone given their medication regimens, medical history, and comorbidities [11,19,20].

In two surveys about pharmacist roles in naloxone dispensing and education, simultaneously administered to patients receiving treatment for substance use disorders and pharmacists in Ohio, patients expressed interest in naloxone-based interventions [21]. Meanwhile, many pharmacists were opposed to facilitating naloxone-based interventions [21]. Some of the same concerns were raised for naloxone consultations as had been identified for consultation with patients about opioid safety and risks. Pharmacists identified barriers to delivering these interventions or services as a lack of training to identify eligible patients, and challenges communicating with patients about the need for naloxone. Pharmacists also identified lack of time, reimbursement, and lack of support from management as barriers to implementing naloxone services [22,23]. Institution-specific guidelines and protocols addressing pharmacist roles, criteria for screening for eligibility, and flowcharts for education and dissemination of naloxone have facilitated identification of patients at risk of overdose, and serve as important resources for pharmacists and other healthcare professionals [24,25]. These resources along with structured programs regarding naloxone use have helped pharmacists successfully dispense naloxone to patients at risk of overdose [18,24,25].

### 4.3. Opioid Storage and Disposal

Pharmacists have a unique opportunity to educate patients on the importance of proper medication disposal and storage and disposal programs available in the community [8,26,27]. Student pharmacists can partner with community officials and businesses to provide safe and appropriate medication disposal [28]. While it is widely understood that medications such as opioids and other controlled substances contribute to environmental pollution when improperly disposed, it has proven hazardous to the population and community health as well [28]. Drug take-back programs have been popular across schools and pharmacies in the country and drug take-back days are helpful in facilitating these activities. Some community pharmacy chains, such as CVS, have installed drug take-back boxes in the pharmacies for patients to dispose of unused or expired prescription medications [29].

### 4.4. PDMP Utilization

Statewide prescription drug monitoring programs (PDMP) are a useful tool for healthcare professionals to track and monitor controlled substance prescriptions [9]. Specific legislative initiatives that support safe opioid prescribing and naloxone use include the 2016 21st Century Cures Act, which awarded funding to improve state PDMPs [30]. The PDMP can serve as a resource for pharmacists to identify patients that might be misusing opioids or those at risk of overdose. Additionally, the 2018 SUPPORT for Patients and Communities Act requires checking the PDMP for Medicare beneficiaries prior to controlled substance prescription. This may prompt further conversations with patients regarding opioid safety [31]. States have varying requirements for PDMP use among pharmacists and prescribers, ranging from voluntary to mandated use [9]. The majority of pharmacists have viewed the PDMP as an objective resource to support clinical decisions, make professional judgements, and prevent diversion and drug abuse [9,32,33,34]. Pharmacists also felt the PDMP helped support patient and prescriber communication regarding suspected drug abuse and helped provide patient education about opioid-specific risks and controlled substance abuse [9,32,35]. While the majority of pharmacists agreed that the PDMP was important in the prescribing and dispensing process, some pharmacists reported barriers to using the PDMP [36]. Pharmacists were less likely to use the PDMP if use was not mandated, if they were unfamiliar with it, didn’t like the user interface, or if no training was provided on using the PDMP platform [9,36]. Some pharmacists also reported challenges in working with prescribers or patients in response to PDMP reports [9]. Factors that facilitated regular use of the PDMP included providing training and education on PDMP use. Integrating the PDMP interface into dispensing software and electronic health records was shown to be particularly powerful, along with mandating use for pharmacists and prescribers at the point of prescribing and dispensing, and the ability to contact the prescriber directly through the PDMP [9,32,36].

### 4.5. Opioid Deprescribing

Deprescribing is a necessary mechanism to reduce the use of inappropriate medications, including medications that cause patient harm or unnecessary adverse effects, medications not providing benefit, or medications with no indication [37]. Deprescribing programs initiated by pharmacists, or that included pharmacists in a multidisciplinary team, have measured successful outcomes such as decreased pill burden or decreased use of inappropriate medications [38,39,40,41,42,43]. However, few guidelines have been developed to guide healthcare professionals in a systematic process to deprescribe or taper inappropriate medications. Systematic processes for deprescribing or tapering inappropriate medications are limited to a few specific classes of medications, and no guidelines exist for opioid discontinuation or tapering [44,45,46,47,48]. Further barriers exist to deprescribing practices in general. Prescribers cite barriers related to lack of time, limited resources to support deprescribing, patient fear, patient withdrawal symptoms, or patient criticism among deprescribing programs involving pharmacists [49,50]. Pharmacists report barriers related to pressures to focus on productivity, rather than clinical interactions or decision-making with patients. Other barriers identified by pharmacists and prescribers include challenges working with patients and caregivers, lack of policies or guidelines specific to deprescribing, and difficulty partnering inter-professionally across health care settings due to lack of shared health information and patients visiting multiple pharmacies [50]. Factors cited by pharmacists and prescribers that would facilitate deprescribing include further involvement of patients and caregivers, staff education, financial incentives, and involvement in initiatives that expand evidence supporting deprescribing practices [50].

### 4.6. Providing Resources for Opioid Misuse and Addiction Treatment

Pharmacists play a key role in recognizing opioid toxicity and preventing diversion of opioids [26]. Compared to prescriber colleagues, pharmacists perceived a larger percentage of patients to be abusing opioids (17% prescribers and 41% pharmacists, respectively) [51]. Pharmacists understand the importance of providing appropriate counseling and resources to such patients but believed that engaging with patients potentially abusing opioids may cause loss of customers. Pharmacists identified regulatory agencies and patients’ family or friends as most likely to influence their willingness to refer patients to resources for opioid misuse [20]. As with the earlier topics, pharmacists cited many of the same barriers to effectively communicating with patients such as lack of confidence, training, and time. Time required for counseling was found to be the most commonly cited control belief [51]. Pharmacists who had greater amounts of addiction-specific education had a higher likelihood of substance abuse counseling and felt more confident about counseling; however, the majority of pharmacists received no addiction education [52]. This suggests that the neurobiological basis for addiction, standards of care, and pain management guidelines are likely not understood by a subset of pharmacists.

## 5. Discussion

Pharmacists are considered gatekeepers for dispensing opioid medications by other health care professionals and in the literature [53]. They are well positioned to facilitate safe opioid use through counseling patients on opioid risks, educating about safe storage and disposal, providing naloxone, participating in deprescribing initiatives, utilizing the PDMP, and connecting patients to resources for addiction treatment [8]. Although these roles for pharmacist services exist, there are opportunities for broader implementation. Pharmacists are interested in providing these services and have identified mechanisms to support safe opioid use among patients as well as barriers to service implementation. Lack of confidence, training and resources, structured guidelines, and limited time were the most commonly cited barriers by pharmacists for providing all the services addressed in this commentary [7,9,14,22,23,24,25,32,50,51,54].

### 5.1. Counseling on Opioid Risks and Safety

Although pharmacists are expected by the Centers For Disease Control and Prevention (CDC) to communicate dependency, overdose, and other opioid risks during patient counseling, pharmacists reported barriers such as lack of confidence, training, and time [7,14,55] * (*The Centers for Disease Control and Prevention (CDC) is a federal agency that conducts and supports health promotion, prevention, and preparedness activities in the United States.). Pharmacists would benefit from additional training and resources in pharmacy school curricula and in practice to facilitate effective counseling for opioid medications. Due to the paucity of research and few initiatives focusing on pharmacist communication for opioid medications, additional research is needed to inform effective training strategies.

### 5.2. Naloxone Dispensing

Pharmacists are recognized as stewards of naloxone dispensing and often stock naloxone in the outpatient setting, but do not necessarily dispense naloxone regularly [22]. Pharmacists expressed a lack of training and confidence to detect patients who would benefit from naloxone and to provide education about the necessity and use of naloxone [22,56]. Targeted training and resources about naloxone dispensing and communication techniques will facilitate further pharmacist involvement in naloxone dispensing and education [18,24].

### 5.3. Education on Opioid Storage and Disposal

Pharmacists recognize their role in informing patients about safe storage and disposal of opioids, yet few initiatives have measured pharmacist improvement, facilitators and barriers to providing such services [27]. There is a need for more evidence-based intervention research about how to help pharmacists offer services to promote safe disposal of opioids [29].

### 5.4. Guidelines and Protocols for PDMP Use and Opioid Deprescribing

Most pharmacists agreed that PDMP was a useful tool, especially when linked to the electronic health record and utilized as a platform to contact prescribers. However, pharmacists also reported challenges when working with patients and prescribers about issues detected in PDMP reports [9,32,36]. Policies and procedures highlighting pharmacists’ roles within the PDMP can help promote more frequent PDMP use and help involve pharmacists in the monitoring process. Beyond the monitoring of prescribed opioids, pharmacists also play an important role in eliminating unnecessary medication when it is inappropriate or no longer needed. Specific algorithms and guidelines would facilitate opioid deprescribing practices in a variety of pharmacy settings. Integrating these into electronic health records would strengthen the intervention even further.

### 5.5. Providing Resources for Addiction Treatment

Pharmacists recognized and valued their role in detecting and communicating with patients potentially misusing prescription opioids [51]. They also expressed lacking confidence and time when talking to patients about opioid abuse [13,51]. Training and resources about addiction treatment should be offered by schools of pharmacy and continuing education programs to facilitate better understanding and identification of resources available to patients in the community.

Overall, training, education, and guidelines for pharmacist roles specific to increasing their readiness to deliver services regarding prescription opioids is warranted for ensuring that pharmacists participate in services that promote safe opioid use among patients. This commentary summarizes services that pharmacists currently offer, assesses factors that affect pharmacists providing these services, suggests strategies for improvement, including areas that should be augmented with education or advocacy in order to ensure enhanced pharmacist services that promote safe opioid use. This commentary has limitations to acknowledge. The scope of this commentary focuses on the opioid crisis and pharmacist services within the U.S., reducing generalizability to other countries. The methodology does not follow a rigorous data extraction and data analysis procedure, but rather includes articles which the authors deemed most applicable and appropriate to include. This paper points to a lack of evidence demonstrating that pharmacist services are directly impacting patient outcomes. All of these services in theory have beneficial effects but it is difficult to find evidence of direct effect of pharmacist services in the literature. Thus, there is need for future research about the impact of pharmacist services in mitigating the opioid crisis.

## 6. Conclusions

This commentary paper is one of its kind to describe current practices and roles of pharmacists and factors that affect pharmacists’ behaviors and attitudes dispensing these services. Pharmacists successfully recognize the importance of their involvement in services to promote safe opioid use. While pharmacists are expected to participate in service provision to mitigate the opioid crisis, it is evident that pharmacists need more targeted training, education, resources, and structured guidelines to increase confidence and self-efficacy in delivering such services.
